# Hydroxychloroquine in nephrology: current status and future directions

**DOI:** 10.1007/s40620-023-01733-6

**Published:** 2023-08-02

**Authors:** Indu Ramachandra Rao, Ashwija Kolakemar, Srinivas Vinayak Shenoy, Ravindra Attur Prabhu, Shankar Prasad Nagaraju, Dharshan Rangaswamy, Mohan Varadanayakanahalli Bhojaraja

**Affiliations:** Department of Nephrology, Kasturba Medical College, Manipal, Manipal Academy of Higher Education, Manipal, Karnataka India 576104

**Keywords:** Antimalarials, Antirheumatic agents, Glomerulonephritis, IgA nephropathy, Immunomodulators, Lupus nephritis, Systemic lupus erythematosus

## Abstract

Hydroxychloroquine is one of the oldest disease-modifying anti-rheumatic drugs in clinical use. The drug interferes with lysosomal activity and antigen presentation, inhibits autophagy, and decreases transcription of pro-inflammatory cytokines. Owing to its immunomodulatory, anti-inflammatory, anti-thrombotic effect, hydroxychloroquine has been an integral part of therapy for systemic lupus erythematosus and lupus nephritis for several decades. The therapeutic versatility of hydroxychloroquine has led to repurposing it for other clinical conditions, with recent studies showing reduction in proteinuria in IgA nephropathy. Research is also underway to investigate the efficacy of hydroxychloroquine in primary membranous nephropathy, Alport’s syndrome, systemic vasculitis, anti-GBM disease, acute kidney injury and for cardiovascular risk reduction in chronic kidney disease. Hydroxychloroquine is well-tolerated, inexpensive, and widely available and therefore, should its indications expand in the future, it would certainly be welcomed. However, clinicians should be aware of the risk of irreversible and progressive retinal toxicity and rarely, cardiomyopathy. Monitoring hydroxychloroquine levels in blood appears to be a promising tool to evaluate compliance, individualize the dose and reduce the risk of retinal toxicity, although this is not yet standard clinical practice. In this review, we discuss the existing knowledge regarding the mechanism of action of hydroxychloroquine, its utility in lupus nephritis and other kidney diseases, the main adverse effects and the evidence gaps that need to be addressed in future research.

**Graphical abstract:**

Created with Biorender.com. HCQ, hydroxychloroquine; GBM, glomerular basement membrane; mDC, myeloid dendritic cell; MHC, major histocompatibility complex; TLR, toll-like receptor

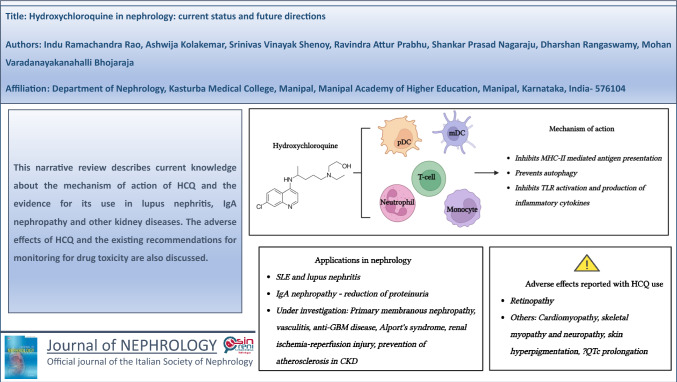

## Introduction

The origin of hydroxychloroquine (HCQ) dates back to the fifteenth century when the medicinal properties of a Peruvian tree bark that was used by the indigenous people to treat fever were discovered by a Jesuit priest [1]. In 1820, quinine was isolated from this so-called “cinchona bark” and the drug soon gained widespread use as a therapeutic and prophylactic agent for malaria [2]. During World War II, soldiers with rheumatic illnesses using quinacrine (a synthetic derivative of quinine) for malaria prophylaxis reported improvement in their symptoms, and this serendipitous discovery paved the way for the use of antimalarials as disease-modifying agents in systemic lupus erythematosus (SLE) and other rheumatological conditions [3]. Antimalarials are now a part of guideline-recommended therapy for patients with SLE and lupus nephritis, in addition to steroids and other immunosuppressive medications [4, 5]. Hydroxychloroquine is the most commonly used drug of its class, when compared to quinacrine, chloroquine, and other quinine derivatives, due to its better tolerability and safety profile. Its use in SLE has been shown to improve remission rates, reduce disease flares, reduce the incidence of cardiovascular and thrombotic events, preserve bone mass, and improve pregnancy outcomes [6].

Despite decades of clinical use, the mechanisms of action of HCQ are still incompletely elucidated. While traditionally believed that HCQ functions by increasing lysosomal pH and thus, inhibiting self-antigen presentation, it is now evident that the immunomodulatory and anti-inflammatory effects of HCQ are much more complex. With a clearer understanding of its action now beginning to emerge, there has been renewed interest in the possible role of HCQ in other kidney diseases. This review aims to provide a comprehensive overview of the mechanism of action of HCQ and the current evidence for its use in lupus nephritis, IgA nephropathy and other kidney diseases. We also discuss the adverse effects of HCQ and the existing recommendations for monitoring drug toxicity.

## Pharmacology

Hydroxychloroquine (C_18_H_26_ClN_3_O) is a 4-aminoquinolone and a racemic mixture of R- and S- enantiomers and is available as 200 mg tablets of hydroxychloroquine sulphate, which is equivalent to 155 mg of HCQ base [7, 8]. The drug has an oral bioavailability of approximately 70%, a large volume of distribution (as high as 47,257 L of blood volume) due to uptake by tissues, and a long half-life of 40–50 days [9, 10]. It undergoes N-dealkylation in the liver by CYP3A4 to the active metabolite desethylhydroxychloroquine and two inactive metabolites, desthylchloroquine and bidesethylchloroquine [11]. Concurrent administration of drugs that induce CYP3A4 enzymes, such as rifampicin and phenytoin, may reduce the blood levels of HCQ, while enzyme inhibitors (ketoconazole, diltiazem, clarithromycin etc.) increase levels [11]. Approximately 40–50% is excreted through the kidneys and so, a dose reduction has been recommended in patients with kidney impairment [4].

Hydroxychloroquine is extensively sequestered in melanin-containing tissues such as skin and retina, which explains the retinopathy and skin hyperpigmentation that has been observed with long-term use, and in other sites such as heart, liver, kidney, brain, and muscles [12]. Although HCQ can cross the placenta and is secreted in breast milk, no toxic effects have been observed and the drug is considered safe in pregnant women and during breastfeeding [13].

## Mechanism of action

### Effect on autoantigen presentation and autophagy

**Fig. 1:**
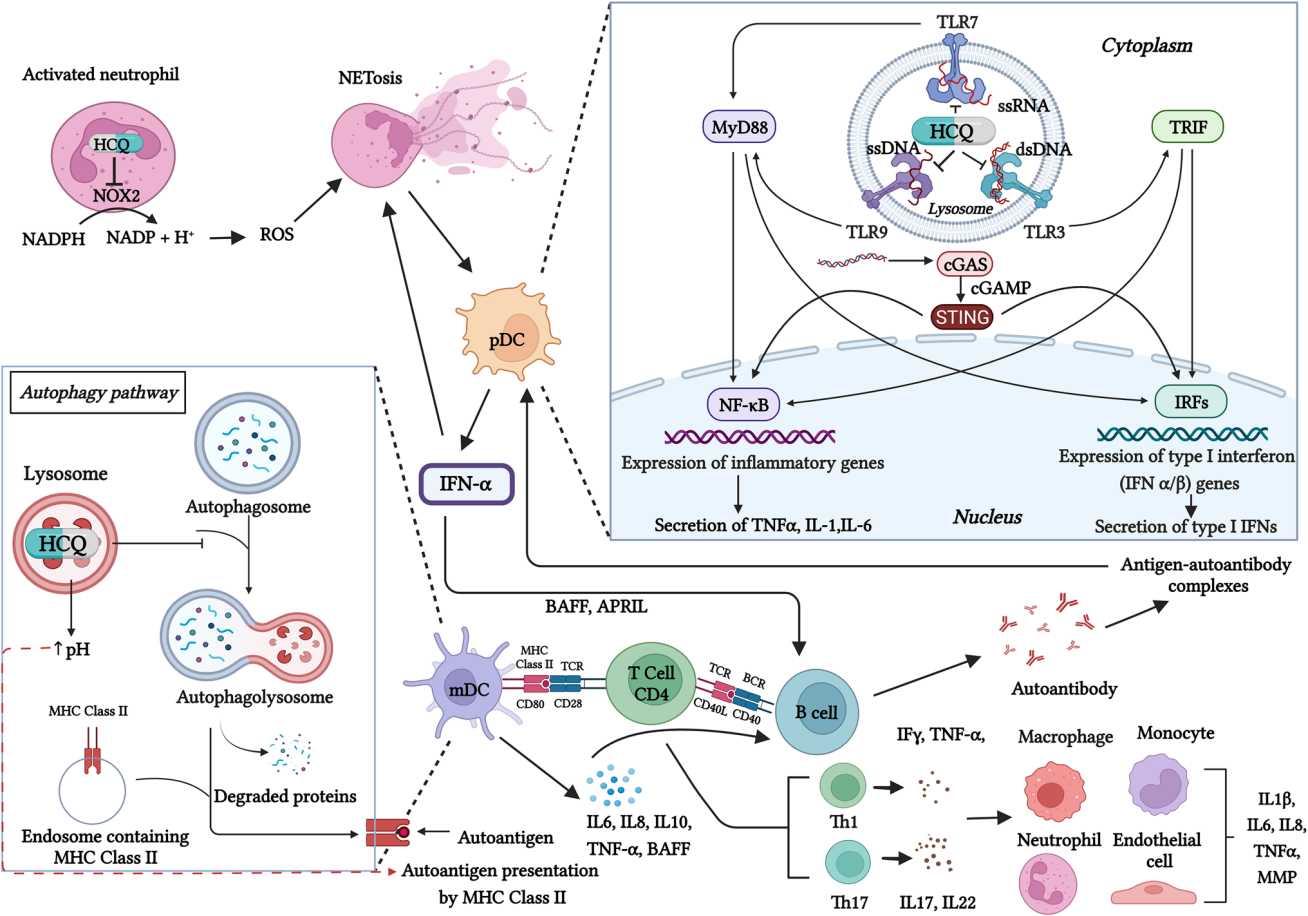
Mechanism of action of HCQ in SLE and lupus nephritis. Hydroxychloroquine accumulates in lysosomes and increases its pH, thereby interfering with antigen processing by lysosomal enzymes. Further, it also prevents autophagy by preventing the fusion of lysosomes with autophagosomes. The net effect is an inhibition of MHC class II-mediated autoantigen presentation by dendritic cells and other antigen-presenting cells to CD4 + T cells. Hydroxychloroquine also prevents the activation of toll-like receptors (TLRs) by nucleic acid ligands and inhibits the cGAS-STING pathway. This inhibits release of type I interferons and other pro-inflammatory cytokines by pDC. Inhibition of endosomal NOX in neutrophils by HCQ leads to reduction of oxidative stress and neutrophil extracellular trap formation (NETosis), which prevents release of IFNα by pDC, along with other cytokines. By its action on TLRs and lysosomes, HCQ also prevents Th1, Th17 and B-cell activation and differentiation. APRIL, A proliferation-inducing ligand; BAFF, B-cell activating factor; BCR, B cell receptor; cGAS, cyclic GMP-AMP synthase; cGAMP, cyclic guanosine monophosphate; dsDNA, double stranded DNA; IFN-α, interferon-alpha; IFN- γ, interferon-gamma; IL, interleukin; IRF, interferon regulatory factor; mDC, myeloid dendritic cell; MHC, major histocompatibility complex; MMP, matrix metalloproteinase; MyD88, myeloid differentiation primary response-88; NET, neutrophil extracellular traps; NOX—NADPH oxidase; NF-kB, nuclear factor-Kappa light chain enhancer of activated B Cells; pDC, plasmacytoid dendritic cell; ROS, reactive oxygen species; ssDNA, single stranded DNA; TCR, T-cell receptor; Th, T-helper; TLR, toll-like receptor; TNFα, tumor necrosis factor-alpha; TRIF, TIR-domain-containing adapter-inducing interferon. The image was created with BioRender.com

The main action of HCQ (and other antimalarials) is believed to occur due to its lysosomotropism, as the Nobel laureate Christian de Duve termed it [8]. Hydroxychloroquine is a lipophilic drug that readily passes through cell membranes and accumulates in lysosomes. Being a weak base, it increases the pH of the lysosome from four to six. This alteration in the milieu results in the inhibition of lysosomal proteases, leading to disturbances in the intracellular processing of antigens, and decreasing binding of antigens to the α and β chains of MHC class II molecules [8] (Fig. 1). Since autoantigens typically have low affinity for MHC class II molecules (compared to non-self-antigens), HCQ has a preferential inhibitory effect on autoimmunity but does not impair immunity against foreign antigens [8, 14].

Another mechanism of action that has received considerable attention is its effect on autophagy. Autophagy is a process by which cellular debris or foreign proteins are degraded by lysosomes, and this plays an important role in the removal of pathogens, antigen presentation, cytokine secretion, and lymphocyte differentiation and activation. Dysregulated autophagy has been implicated in the pathogenesis of SLE and other autoimmune diseases [15]. By its effect on lysosomal pH, HCQ prevents the fusion of lysosomes with autophagosomes (double-membrane vesicles that engulf proteins targeted for degradation) and functions as a potent autophagy inhibitor (Fig. 1) [16]. However, it is important to note that recent animal studies have found that autophagy plays a pivotal role in the maintenance of podocyte and endothelial integrity, and that deficient autophagy leads to podocyte injury and promotes a pro-inflammatory and pro-atherogenic milieu in the endothelium [17, 18]. Therefore, the net effect of HCQ-mediated autophagy inhibition needs to be further elucidated.

### Effect on toll-like receptor signaling

Toll-like receptors are pattern-recognition receptors that play a crucial role in innate immunity due to their ability to recognize a variety of pathogen-associated molecular patterns. Toll-like receptor activation by binding to its ligand causes dendritic cells to produce interferon-α (IFN- α) and stimulates B cells to produce antibodies and cytokines and to upregulate their expression of costimulatory molecules. Toll-like receptors-3,-7,-8, and -9 have been implicated in the pathogenesis of SLE [19]. These TLRs are intracellularly located on endosomes and lysosomes and recognize nucleic acids, both self and foreign. Hydroxychloroquine inhibits TLR function by two postulated mechanisms; firstly, it can interfere with TLR activity indirectly by its effect on endosomal/lysosomal pH and secondly, it can directly bind to nucleic acids thereby inhibiting TLR-nucleic acid binding (Fig. 1) [20].

### Effect on cytokine production

Hydroxychloroquine indirectly reduces the production of various cytokines such as interleukin-1 (IL-1), tumour necrosis factor-α (TNF-α), and interferon (IFN)-γ by macrophages, monocytes, and plasmacytoid dendritic cells [21]. Through its effect on TLR signaling, production of type I interferons such as IFN-α and -β are also reduced. Hydroxychloroquine also inhibits cyclic GMP-AMP synthase (cGAS) activity [22] which, when activated through nucleic acid ligands, leads to a stimulator of IFN gene (STING)-mediated transcription of IFN-α [23] (Fig. 1). The activity of endosomal NAPDH-oxidase (NOX) is also inhibited leading to the reduction of IL-8 and TNF-α [24]. Studies have also found a reduction in Th17-related cytokines (IL-6, IL-17 and IL-22) with HCQ use, possibly by rebalancing the Th17/Treg ratio through its effect on autophagy [25, 26].

### Other effects

Hydroxychloroquine blocks the release of calcium from the endoplasmic reticulum into the cytoplasm, interfering with calcium-dependent signaling, an important mechanism for T- and B-cell activation [27]. It also induces Fas-mediated apoptosis [28]. It exerts an anti-thrombotic effect by inhibiting platelet aggregation and preventing the binding of anti-phospholipid antibody (APLA)- β2-glycoprotein I (β2-GPI) complex to phospholipid bilayers [29]. Phospholipase A2 is also inhibited by HCQ, thereby altering arachidonic acid metabolism and reducing the production of prostaglandins and leukotrienes [30]. A reduction in serum levels of B-cell activating factor (BAFF), a survival factor for B-cells, has been reported with HCQ [31]. The drug also inhibits matrix metalloproteinase-9 (MMP-9) and tissue inhibitor of metalloproteinases-1 (TIMP-1) [32].

## HCQ in lupus nephritis

Lupus nephritis, which occurs in 20–60% of SLE patients over their lifetime, is one of the most severe forms of SLE [33]. It is associated with high morbidity and mortality and is the most common cause of disease-related mortality in SLE [34, 35]. The first evidence of HCQ efficacy in SLE patients with kidney involvement came from the Lupus in Minorities: Nature versus nurture (LUMINA) cohort, a prospective multi-ethnic cohort of SLE patients, in which it was observed that HCQ administration was associated with a lower risk of lupus nephritis [36, 37]. Fessler et al. reported that, in this cohort of 518 patients, the occurrence of nephritis was higher among those not taking HCQ (*P* < 0.0001) [37]. It was also found that the severity of renal involvement may be attenuated with HCQ, with a lower frequency of Class IV glomerulonephritis among HCQ users, compared to those who were not on HCQ (9.9 vs 33.3%, *P* = 0. 0003) [38].

Further, in those with lupus nephritis, HCQ may improve renal remission and reduce risk of renal flares. A study of 29 patients with Class V lupus nephritis found higher complete renal remission rates at one year in patients on HCQ, compared to those not on HCQ (64 vs. 22%; *P* = 0.036) [39]. This finding was confirmed by Mejia-Vilet et al. who reported that complete remission rates were more than twofold higher in Class V nephritis patients treated with antimalarials than among those not on antimalarials (HR 2.46, 95% CI 1.08–5.64; *P* = 0.032) [40]. Data from the Spanish Society of Rheumatology Registry of Patients with Systemic Lupus Erythematosus (RELESSER) registry, that also included patients with proliferative forms of lupus nephritis, suggested that HCQ users had a 60% higher probability of achieving complete response, compared to non-users (OR 1.61, 95% CI1.10–2.36; *P* = 0.014) [41]. A retrospective cohort study of 60 patients with lupus nephritis and renal impairment (eGFR < 60 mL/min/1.73 m^2^) found that renal recovery at 6 months was more likely among those on HCQ (OR 3.891, 95% CI 1.19–12.65; *P* = 0.024) [42]. More recently, a randomized placebo-controlled trial of 60 children with Class III/IV lupus nephritis treated with mycophenolate mofetil and steroids found that a higher number of patients in the HCQ arm achieved remission (complete or partial) at 12 months, compared to the placebo group (97 vs. 83%, *P* = 0.002) [43]. The Canadian Hydroxychloroquine Study Group, in a landmark randomized control trial of 47 patients with quiescent lupus, found a reduced risk of renal flares in the HCQ-continuation group, as compared to the HCQ-withdrawal group (RR 0.26; 95% CI 0.03–2.54) at a follow-up of three years [44].

Hydroxychloroquine may also delay CKD progression and reduce risk of end-stage kidney disease (ESKD). Data from the LUMINA cohort suggested that HCQ was associated with a reduction in risk of renal damage, defined as eGFR < 50%, proteinuria ≥ 3.5 g/day lasting for at least 6 months, and/or ESKD (HR 0.12, 95% CI 0.02–0.97; *P* = 0.0464) [38]. Sisó et al. reported that the proportion of patients with high creatinine values of > 4 mg/dL and ESKD was lower among those who received antimalarials prior to the development of nephritis, as compared to those who did not (2 vs 11%; *P* = 0.044) and (2 vs 11%; *P* = 0.029), respectively [45]. Pokroy-Shapira et al. too reported a lower risk of CKD (stage 3 and above) with HCQ use (HR 0.40; 95% CI 0.20–0.90, *P* = 0.02) in a single-center study in Israel [46]. Antimalarial use was also associated with improved renal survival in a retrospective study of patients with membranous lupus nephritis (*P* = 0.007) [47]. Further, an analysis of lupus nephritis patients from the Aspreva Lupus Management Study (ALMS) found that lack of treatment with antimalarials was associated with a two-fold higher likelihood of treatment failure, defined by a composite of death, ESKD, sustained doubling of serum creatinine, renal flare and requirement of rescue therapy [48]. Kidney biopsy studies have also demonstrated a lower degree of tubulointerstitial scarring among lupus nephritis patients on HCQ [49]. In contrast, Wu et al., in a retrospective analysis of a nation-wide cohort of SLE patients, reported no significant difference in the risk of developing CKD among those using HCQs for > 90 days, compared to those who used it for < 90 days (HR 1.295, 95% CI 0.40–4.25) [50].

There is also evidence to suggest that antimalarials reduce the risk of infections in patients with lupus nephritis. Sisó et al. reported fewer infections (11 vs. 29%, *P* = 0.006) in a retrospective cohort study of lupus nephritis patients [45]. Feldman et al. in a study of 33,565 Medicaid beneficiaries with SLE, with a sub-cohort of 7113 lupus nephritis patients, reported a reduced risk of serious infections requiring hospitalization in HCQ users, compared to never-users both in the overall cohort (HR 0.73; 95% CI 0.68–0.77) and in the lupus nephritis cohort (HR 0.78; 95% CI 0.71–0.87) [51]. Similarly, findings from the Grupo Latino Americano De Estudio del Lupus (GLADEL) cohort study (HR 0.69; 95% CI 0.48–0.99; *P* = 0.044) also support the protective effect of HCQ on serious infections [52].

Additionally, HCQ may be associated with lower mortality rates in patients with lupus nephritis, as reported by Sisó et al. (2 vs 13%,* P* = 0.029) [45]. A reduction in all-cause mortality has also been reported by Mok et al. (HR 0.58, 95% CI 0.34–0.99; *P* = 0.048) and Zheng et al. (HR 0.20; 95% CI 0.05–0.82, *P* = 0.026) [53, 54].

A beneficial effect of HCQ on lipid profile has also been reported, with lower levels of total cholesterol and LDL- cholesterol reported in HCQ users, compared to non-users in a Chinese cohort study [55]. Similarly, in the LUMINA cohort, those on HCQ were found to have lower mean LDL levels (110.4 ± 48.0 vs. 138.8 ± 95.8, *P* = 0.0155), compared to those not on the drug [38].

A prospective, multicenter study of pregnant women with lupus nephritis found that HCQ users had an 85% reduction in the odds of having a small-for-gestational-age baby (OR 0.15, 95% CI 0.03–0.77; *P* = 0.0023) [56]. Other benefits including a lower frequency of hypertension (32 vs 50%, *P* = 0.027) and thrombotic events (5 vs. 17%, *P* = 0.04) were reported by Sisó et al., although there was no reduction in the risk of malignancy (OR 0.23, 95% CI 0.01–4.30), stroke (OR 1.44, 95% CI 0.46–4.55) or ischemic heart disease (OR 1.93, 95% CI 0.41–9.09) [45].

## HCQ in IgA nephropathy

**Fig. 2:**
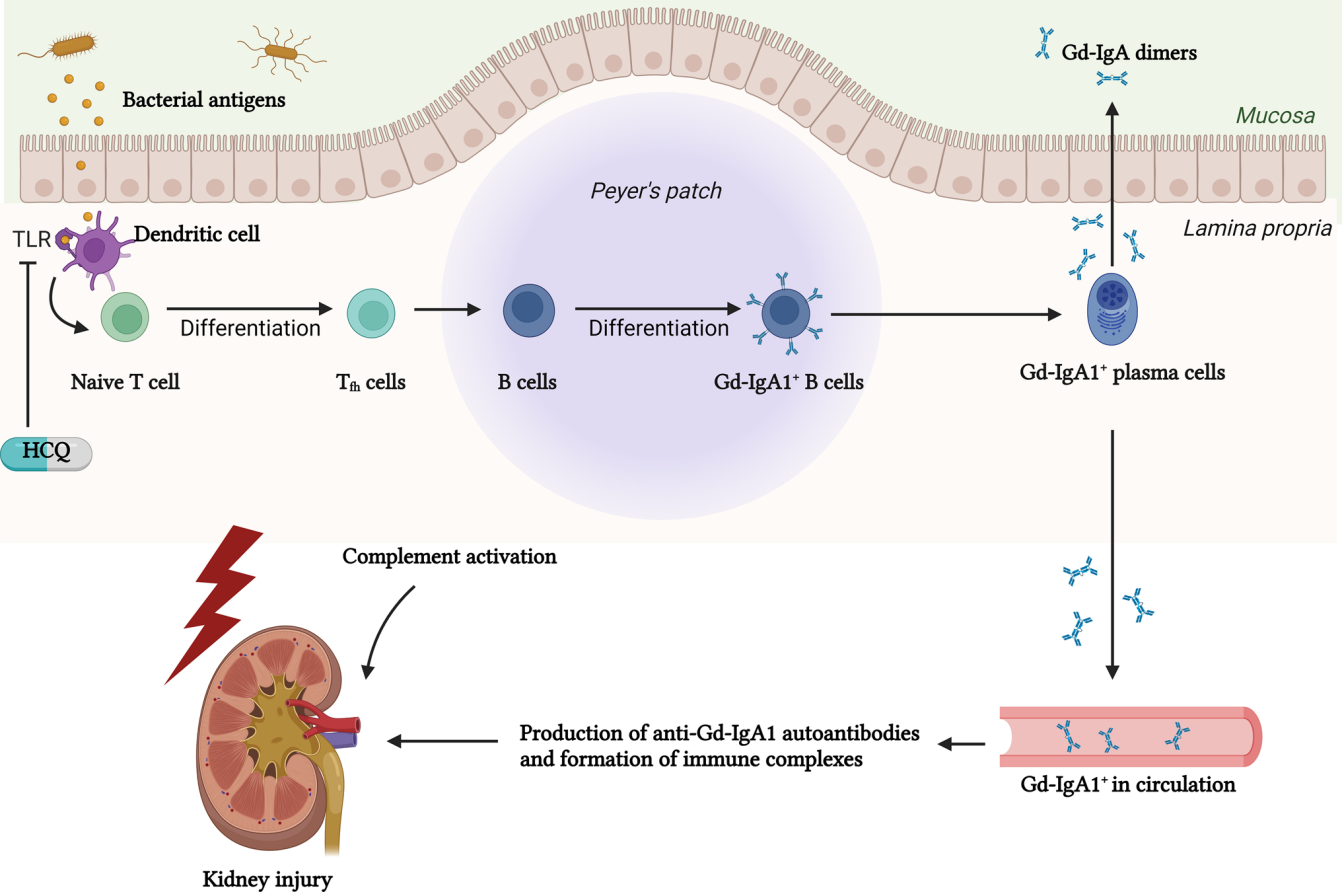
Mechanism of action of HCQ in IgA nephropathy. Created with BioRender.com. Gd, galactose deficient; HCQ, hydroxychloroquine; TLR, toll-like receptor

Defective glycosylation of IgA1 leading to increased circulating levels of galactose-deficient IgA1 (Gd-IgA1) is the key factor in the pathogenesis of IgA nephropathy [57]. This leads to the formation of IgG and IgA autoantibodies against Gd-IgA1, and the immune complexes thus formed deposit in the glomerular mesangium leading to renal injury. It is proposed that HCQ, by its targeting of TLR signaling and reduction of levels of cytokines such as IL-6, IFN-α and TNF-α, may be effective in attenuating renal damage in IgA nephropathy (Fig. 2) [58].

Data from observational studies and a single randomized control trial (RCT) indicate that HCQ may have a role as an adjuvant antiproteinuric therapy for IgA nephropathy in patients who have persistent proteinuria despite optimal supportive therapy. A pilot study of 28 patients with IgA nephropathy and proteinuria of 0.5–2.0 g/day found that those on HCQ (in addition to losartan) were more likely to achieve a 50% reduction of proteinuria at the end of 24 weeks of treatment compared to those on losartan alone (42.9 vs. 14.3%, *P* = 0.004) [59]. Similarly, Yang et al., in a propensity-score matched retrospective cohort study, reported that more than two-thirds of patients on HCQ had a reduction in proteinuria by 30% or more within 6 months, compared to 45.6% of those on RAS inhibitors alone [60]. Further, the authors noted that this effect on proteinuria reduction persisted at 24 months of follow-up, with no significant decline in eGFR or serious adverse events during this period.

Retrospective data showed similar reductions in proteinuria between patients treated with HCQ and those on corticosteroids, suggesting that HCQ may serve as an alternate treatment for IgA nephropathy in patients wishing to avoid side-effects related to steroid use [61, 62]. Bagchi et al. reported that, of 38 patients with IgA nephropathy and persistent proteinuria of > 1 g/day despite at least 6 months of conservative therapy with RAS inhibitors, 21 achieved remission with the addition of HCQ at the end of a follow-up period of 6 months [63]. Interestingly, the authors also observed a relapse of proteinuria in 20% of the patients on HCQ discontinuation, suggesting that long-term use of HCQ may be needed.

More recently, Liu et al. randomized sixty subjects with IgA nephropathy, all of whom had an eGFR of > 30 ml/min/1.73 m^2^, proteinuria of 0.75–3.5 g/day and were on maximal tolerated doses of RAS inhibitors, to receive either HCQ or placebo, and found a significant reduction in proteinuria in the HCQ group at 6 months, compared to the placebo group (percentage reduction of − 48.4 vs. 10%,* P* < 0.001) [64]. Half of those in the HCQ group had a 50% proteinuria reduction, compared to 14.8% in the placebo group at 6 months (*P* = 0.006). At the end of 6 months, the trial was stopped and therefore, data on long-term efficacy of HCQ are lacking [64]. This is especially unfortunate since, given the long half-life of HCQ, it takes roughly 6 months for 96% of steady state levels to be achieved and so it would not be unjustified to expect further reductions in proteinuria on longer follow-up [7]. On the other hand, the longer the duration of HCQ administration, the greater the risk of retinal toxicity and other adverse events. It is also important to keep in mind that proteinuria is only a surrogate outcome and hence, it is still unclear if HCQ use in IgA nephropathy can lead to reduction in ESKD and other hard outcomes.

## Other potential indications for HCQ in nephrology

As with IgA nephropathy, HCQ may also exert an antiproteinuric effect in patients with primary membranous nephropathy. A recent prospective cohort study of 126 patients with phospholipase A2 receptor (PLA2R)-associated membranous nephropathy found that a higher proportion of patients receiving a combination of HCQ and renin angiotensin system inhibitors attained a > 30% proteinuria reduction at 6 months, compared to those on renin angiotensin system inhibitors alone (57.5 vs. 28.9%, *P* = 0.002). The proportion of patients requiring initiation of immunosuppressive therapy was also lower in the HCQ plus renin angiotensin system inhibitors group (25 vs. 45.8%, *P* = 0.027). On long-term follow-up, higher rates of clinical remission were noted at two years in the HCQ group (62.5 vs 38.6%, *P* = 0.013). Importantly, it was found that a greater reduction in anti-PLA2R antibody levels was seen with HCQ therapy, implying that its immunomodulatory effect might be responsible for the observed benefit [65]. Ning et al. recently reported a membranous nephropathy patient with concomitant diabetic nephropathy who was successfully treated with a combination of HCQ and rituximab, although in this case the individual contribution of HCQ to the observed therapeutic effect cannot be ascertained [66]. There is a need for well-conducted RCTs to confirm these findings.

It has been postulated that HCQ may be effective in the treatment of systemic vasculitis based on mechanistic considerations, although evidence remains limited. Casian et al. reported symptomatic improvement in fatigue, joint pain, and cutaneous manifestations in sixteen of twenty-six patients with ANCA-associated vasculitis (AAV) treated with a daily dose of 200 mg of HCQ in a retrospective study. The authors also reported that relapses were less frequent and a third of patients were able to reduce steroid doses [67]. Hydroxychloroquine in ANCA Vasculitis Evaluation (HAVEN) is an ongoing multicenter RCT that will attempt to study the effect of the addition of HCQ to maintenance therapy, compared to placebo on disease activity as assessed by the Birmingham Vasculitis Activity Score (BVAS) [68]. The effect of HCQ on renal outcomes in AAV still needs to be explored.

Hydroxychloroquine is also being studied as a potential therapy for those with X-linked Alport’s syndrome (XLAS). It has been postulated that the benefit of HCQ in XLAS may be mediated by its effect on TLRs, although mechanistic data are lacking [69]. Sun et al., in a retrospective case series, reported a significant reduction in microscopic hematuria and/or proteinuria in eight children with XLAS [69]. A phase 2 single-center study is ongoing in China which will randomize 50 participants with XLAS aged between 3 and 18 years to receive either HCQ at a dose of 6.5 mg/kg/day, in addition to angiotensin-converting enzyme inhibitors or standard care (angiotensin-converting enzyme inhibitors alone) for 6 months [70]. Changes in urinary erythrocyte count, proteinuria, and eGFR will be assessed at week 48 in this study.

Data from preclinical studies also indicate a possible anti-inflammatory effect in anti-GBM nephritis. Torigoe et al. found that in rats with anti-GBM nephritis, administration of HCQ improved renal function, and reduced both proteinuria and microscopic hematuria [71]. Hydroxychloroquine use also resulted in improvement in renal histology, with a significant reduction in fibrinoid necrosis and extracapillary proliferation seen in HCQ-treated rats compared to controls. It was also found that the phosphorylation of Jun N-terminal kinase (JNK) and p38 was significantly lower in rats receiving HCQ. Consequently, the authors postulated that the beneficial effects of HCQ in anti-GBM disease may be mediated through its effect on JNL/p38 mitogen-activated protein kinase (MAPK) signaling [71].

It has been suggested that HCQ may exert a nephroprotective effect in ischemia/reperfusion injury based on animal model studies. Tang et al. found that HCQ reduced renal interstitial infiltration by macrophages and neutrophils, decreased levels of pro-inflammatory cytokines, and mitigated the rise in serum creatinine in mice subjected to ischemia/reperfusion injury, compared to controls [72]. Similarly, Zheng et al., in another mouse model study, found that HCQ inhibited macrophage activation and MAPK signaling via its TLR-9 in a dose-dependent manner and attenuated renal fibrosis [73].

The efficacy of HCQ for the prevention of cardiovascular disease and atherosclerosis in CKD is also under investigation. [74] A phase 2 RCT is currently evaluating the effect of HCQ on atherosclerosis, inflammation, and vascular stiffness in patients with CKD. [75]

## Adverse effects

### Retinopathy

Retinopathy is a major dose-limiting toxicity of hydroxychloroquine that can result in irreversible visual impairment or blindness. While believed to be a rare side-effect, the use of more sensitive screening modalities has resulted in higher detection rates and thus in an increased overall prevalence [76]. The clinical appearance is typically described as a bilateral “bull’s-eye” maculopathy, a condition characterized by a perifoveal ring of retinal pigment atrophy, surrounded by normal epithelium. This has been described as a late finding and patients may progress despite discontinuation of the drug. Since the fovea is spared early on, visual acuity usually stays unaffected and the disorder can only be identified on objective examination [77]. As retinopathy progresses, reduced visual acuity, decreased peripheral and poor night vision may ensue. It is unclear how exactly the retinopathy occurs; however, it is hypothesized that hydroxychloroquine induces retinal pigment epithelium degradation and interferes with the ability of lysosome to degrade photoreceptor outer segments [78].

### Cardiotoxicity and myopathy

Cardiotoxicity is a rare yet dangerous side effect of hydroxychloroquine. As with retinopathy, cardiomyopathy due to HCQ is time- and dose-dependent. Hydroxychloroquine inhibits lysosomal enzymes, one of which is alpha-galactosidase A- the enzyme implicated in the pathogenesis of Fabry disease [79]. Inhibition of this enzyme causes the accumulation of globotriaosylceramide in cardiac, skeletal, and smooth muscles and vascular endothelium, leading to infiltrative cardiomyopathy. Patients may present with bi-atrial, concentric ventricular hypertrophy, valvular defects, pulmonary arterial hypertension, as well as conduction abnormalities, and heart failure [80]. Unlike retinopathy, reversibility following withdrawal of antimalarials has been reported [80, 81].

There are also case reports of proximal myopathy due to HCQ, with muscle biopsies showing vacuolar changes and the presence of curvilinear bodies on electron microscopy, with the latter finding considered to be specific for HCQ-associated myopathy [82].

Recent studies have reported prolongation of QT_c_ intervals with HCQ use, however, data are conflicting [83–85]. An analysis of the U.S. Food and Drug Administration's Adverse Event Reporting System suggested that HCQ use was not associated with a safety signal for QT_c_ prolongation or torsades de points [86]. It appears that HCQ-associated QT_c_ prolongation is more often seen in the setting of COVID-19 infection, rather than in rheumatic diseases [85, 87].

### Other side effects

Skin hyperpigmentation, ranging from blueish grey to dark purple, has been reported in up to 25% of patients, starting after as early as 3 months of HCQ therapy. The majority of lesions affect sun-exposed areas and lower extremities, although they can also affect the oral mucosa and nail bed [88]. Gradual resolution with HCQ discontinuation has been reported [89].

Non-specific gastrointestinal symptoms like nausea, vomiting, and diarrhea have been reported, with a reported frequency of as high as 50% [90]. Drug-associated liver injury is a very rare complication, with isolated reports of severe hepatitis in HCQ users [91].

Presence of zebra bodies in the kidney, similar to those seen in Fabry disease, has been reported, caused by HCQ-induced phospholipidosis [92]. The clinical significance of this finding is unclear, and cessation of HCQ has not been recommended.

## Dosing of HCQ and monitoring of therapy

The commonly used dose for SLE and lupus nephritis is 200–400 mg/day of HCQ salt (155–310 mg base), given as a single dose or two divided doses. At a dose of 4–5 mg/kg/day, the reported prevalence of retinopathy is < 2% in the first ten years of use, increasing to 20% after 20 years of use [93]. Based on these data, major rheumatological and ophthalmological guidelines have recommended that the optimal daily dose of HCQ be ≤ 5 mg/kg/day (of actual body weight) [4, 94]. Since existing literature on HCQ in lupus nephritis is based on higher doses, the clinical implications of lower doses is yet unknown. Notably, a recent study reported higher flares with an HCQ dose of 5 mg/kg/day or lower, compared to higher doses (adjusted OR 1.98, 95% CI 1.03–3.79); however, the retrospective nature of the study and the lack of data regarding adherence to the drug preclude a definite conclusion [95]. At present, the 2021 KDIGO guidelines suggest an HCQ starting dose of 6.5 mg/kg of ideal (not actual) body weight initially, followed by a maintenance dose of 4–5 mg/kg of ideal body weight [5].

Another area of debate is the need for dose modification for patients with kidney impairment. Although not recommended by drug manufacturers, based on the fact that a significant proportion of the drug is excreted through the kidneys, the KDIGO guidelines recommend a dose reduction of at least 25% for those with an eGFR of < 30 mL/min/1.73 m^2^, while the EULAR/ERA-EDTA guidelines recommend a 50% dose reduction [5, 96].

**Fig. 3:**
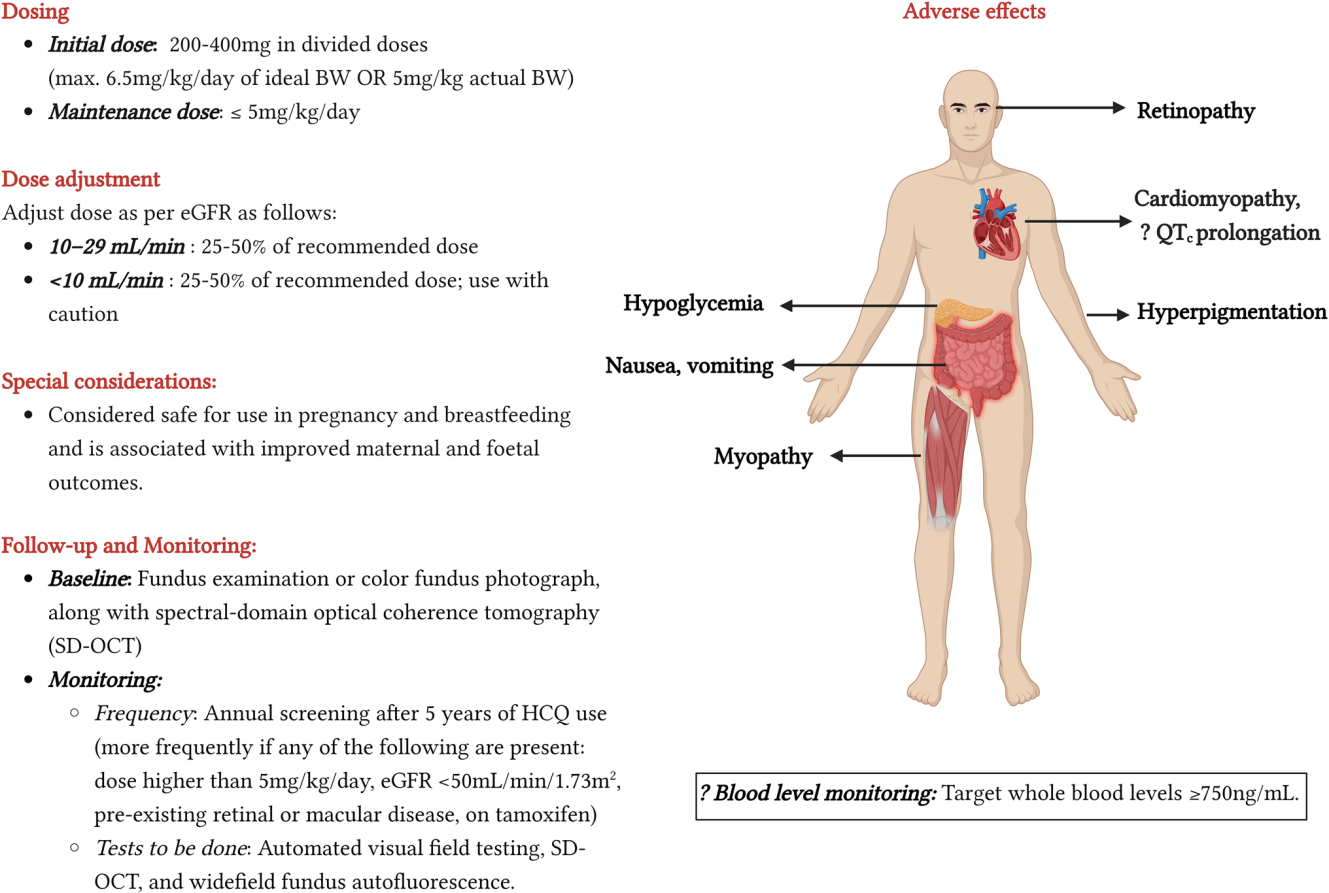
Practical considerations for the use of HCQ. Created with BioRender.com. BW, body weight; eGFR, estimated glomerular filtration rate

Fundus examination or color fundus photography, along with spectral-domain optical coherence tomography (SD-OCT) has been recommended at baseline, followed by annual screening after 5 years of use **(**Fig. 3.) [97]. Patients on higher doses of HCQ, having renal impairment with an eGFR of < 50 mL/min/1.73 m^2^, pre-existing retinal/macular disease or are taking tamoxifen are at higher risk of retinopathy and would require more frequent ophthalmological screening [93, 97] Annual screening should ideally include automated visual field testing, SD-OCT, and widefield fundus autofluorescence (Fig. 1).

There is a growing body of evidence regarding the clinical utility of monitoring blood levels of HCQ. Petri et al. reported that higher whole blood HCQ levels, measured by liquid chromatography-tandem mass spectroscopy, were associated with a higher risk of retinopathy (*P* = 0.0124) [98]. Studies have also found that the lower the HCQ levels, the higher the disease activity in SLE, with a systematic review reporting a 58% lower risk of active lupus with HCQ levels of ≥ 750 ng/mL [99]. Blood level monitoring may also aid in identifying defaulters, with Costedoat-Chalumeau et al. suggesting a cut-off of < 200 ng/mL for severe non-adherence [100].

## Evidence gaps, future directions and conclusions

Treatment with HCQ (or other equivalent antimalarials) in lupus nephritis is a Class I recommendation as per the KDIGO guidelines and indeed most nephrologists include antimalarials as background therapy for lupus nephritis. However, the existing data for HCQ use in lupus nephritis are mostly observational in nature and based on low-quality evidence (Table 1). It is unlikely, however, that an RCT would ever be conducted since, given the large effect sizes for improved kidney and patient outcomes observed in observational studies and the evidence of increased flares on HCQ withdrawal, such a study would be considered unethical.

**Table 1:** HCQ and other antimalarials in SLE and lupus nephritis

S. No.	Author (year)	Study type	Participants, no	Summary of results
*Renal effects*				
A. Reduction of proteinuria				
1.	Kasitanon et al. [39]	Retrospective cohort	29 patients with Class V LN	Patients on HCQ were more likely to achieve remission within 12 months, compared to those not on HCQ (64 vs. 22%, *P* = 0.036)
2.	Mejia-Vilet et al. [40]	Retrospective cohort	60 patients with Class V LN	Patients treated with antimalarials were more likely to achieve complete remission compared to those not on antimalarials (HR 2.46, 95% CI 1.08–5.64; *P* = 0.032)
3.	Galindo-Izquierdo et al. [41]	Retrospective cohort	3575 SLE patients (1092 patients with LN)	Those on antimalarials had higher complete remission rates (OR 1.61, 95% CI 1.10–2.36; *P* = 0.014)
4.	Gheet et al. [43]	RCT	60 children with Class III/IV LN	At 12 months, the cumulative probability of partial and complete remission was 40% and 60% in the HCQ arm, compared to 53.3% and 36.7% in the placebo arm (*P* = 0.002)
B. Development of lupus nephritis				
1.	Fessler et al. [37]	Prospective cohort (LUMINA)	518 SLE patients	HCQ non-users had a higher risk of major organ involvement, such as nephritis (P < 0.0001) and central nervous system disease (*P* < 0.003)
2.	Tsang-A-Sjoe et al. [102]	Prospective cohort	190 SLE patients	Non-use of HCQ was associated with higher risk of LN (*P* = 0.024)
3.	Galindo-Izquierdo et al. [41]	Retrospective cohort	3575 SLE patients (1092 patients with LN)	Patients on antimalarials had lower frequency of lupus nephritis (OR 0.58, 95% CI 0.48–0.70; *P* < 0.001)
C. ESKD/CKD progression				
1.	Sisó et al. [45]	Retrospective cohort	206 patients with LN	Patients on HCQ prior to diagnosis of LN had a lower frequency of creatinine values > 4 mg/dL (2 vs 11%, *P* = 0.029) and end-stage kidney disease (2 vs 11%, *P* = 0.044) in comparison with those never treated with HCQ
2.	Pons-Estel et al. [38]	Prospective cohort	256 patients with LN	Renal damage defined as eGFR < 50 mL/min, 24 h proteinuria ≥ 3.5 g and/or end-stage renal disease was less likely in those on HCQ (HR 0.12, 95% CI 0.02–0.97; *P* = 0.046)
3.	Okpechi et al. [47]	Retrospective cohort	42 patients with Class V LN	Patients who received HCQ had better renal survival compared with those who did not (*P* = 0.007)
4.	Pokroy-Shapira et al. [46]	Prospective cohort	256 SLE patients	Lower risk of CKD (stage 3 and above) was found with HCQ use (HR 0.4; 95% CI: 0.2–0.9, *P* = 0.02)
5.	Kwon et al. [49]	Retrospective study	52 LN patients	Use of HCQ, both length of treatment with HCQ (adjusted OR 0.974, 95% CI 0.951–0.998, *P* = 0.036) and cumulative dose of HCQ (log transferred value) (adjusted OR 0.485, 95% CI 0.262–0.896, *P* = 0.020) were inversely associated with aggravation of tubulointerstitial damage
6.	Lee et al. [42]	Retrospective longitudinal cohort study	90 patients with LN	On multivariate analysis, hydroxychloroquine use [odds ratio (OR) = 3.891, 95% confidence interval (CI) 1.196–12.653, *P* = 0.024], prolonged LN (OR = 0.926, 95% CI 0.874–0.981, *P* = 0.009) and high-grade tubular atrophy (OR = 0.451, 95% CI 0.208–0.829, *P* = 0.013) were associated with renal function recovery. During follow up, 25 patients reached end-stage renal disease (ESRD)
7.	Wu et al. [50]	Retrospective analysis	783 SLE patients	No significant difference in the risk of developing CKD among those using HCQ for > 90 days, compared to those who used it for < 90 days (HR 1.295, 95% CI 0.40–4.25)
D. Renal flare				
1.	Tsakonas et al. [44]	RCT	47 patients with quiescent SLE	HCQ-continuation group, showed a reduced risk of renal flares as compared to the HCQ-withdrawal group (RR 0.26; 95% CI 0.03–2.54) at a follow-up of three years
*Extra-renal benefits*				
A. Mortality				
1.	Alarcon et al. [103]	Nested case–control study within the LUMINA cohort	608 SLE patients	HCQ users had lower mortality, compared to non-users (OR 0.13, 95% CI 0.05–0.30)
2.	Sisó et al. [45]	Retrospective cohort	206 patients with LN	HCQ exposure prior to LN diagnosis was associated with lower mortality rates (2 vs 13%,* P* = 0.029)
3.	Shinjo et al. [104]	Prospective cohort	1480 SLE patients	Antimalarial use was associated with lower mortality (HR 0.62, 95% CI 0.39–0.99)
4.	Zheng et al. [54]	Retrospective cohort	491 patients with LN	HCQ improved survival of patients with LN (HR for mortality 0.20; 95% CI 0.05–0.82, *P* = 0.026]
5.	Mok et al. [53]	Prospective cohort	803 SLE patients	The use of HCQ had a survival benefit in patients with SLE (HR for mortality 0.59, 95% CI 0.37–0.93)
B. Infections				
1.	Sisó et al. [45]	Retrospective cohort	206 patients with biopsy-proven LN	Fewer infections among patients using HCQ before diagnosis of LN, compared to those not on HCQ (11 vs. 29%, *P* = 0.006)
2.	Feldman et al. [51]	Retrospective cohort	33,565 patients with SLE (7113 with LN)	HCQ users had a reduced risk of infection as compared to never users (HR 0.7, 95% CI 0.68–0.77)
3.	Herrinton et al. [105]	Retrospective cohort	3030 patients with SLE	On comparison with those on HCQ alone, the HR for infection was 3.9 (95% CI 1.7–9.2) for those on GC at a dose of ≤ 15 mg/day without HCQ (14 infections/252 patient years), while it was 0.0 (0 infections/128 patient-years) for those on a combination of HCQ and GC
4.	Rúa-Figueroa et al. [106]	Retrospective cohort	3658 SLE patients	Duration of HCQ use (months) was associated with lower risk of infections (HR = 0.99, 95% CI 0.997–0.999)
5.	Pimentel-Quiroz et al. [52]	Prospective cohort	1243 SLE patients	HCQ use was protective against serious infections (HR 0.69; 95% CI 0.48–0.99; *P* = 0.044)
6.	Yeo et al. [107]	Case–control	406 SLE patients; 58 Pneumocystis pneumonia (PCP) cases and 348 non-PCP controls matched by age, sex and disease-duration	Use of higher 3 months cumulative dose of HCQ was associated with a reduced risk of PCP (OR 0.69, 95% CI 0.21–2.24)
C. Thrombosis				
1.	Sisó et al. [45]	Retrospective cohort	206 patients with biopsy-proven LN	Lower frequency of thrombosis among patients on HCQ (5 vs. 17%, *P* = 0.04)
2.	Mok et al. [108]	Prospective cohort	272 SLE patients	Patients on HCQ had fewer thrombotic complications (OR 0.17, 95% CI 0.07–0.44; *P* < 0.0001)
3.	Ruiz-Irastorza et al. [109]	Prospective cohort	232 SLE patients	HCQ was protective against thrombotic complications (HR 0.28, 95% CI 0.08–0.90)
4.	Petri et al. [110]	Prospective cohort	739 patients with SLE	Thrombosis rates were reduced by 13% for every 200 ng/ml increase in mean HCQ blood level (RR 0.87, 95% CI 0.76–1.00), *P* = 0.056)
D. Lipid profile				
1.	Wallace et al. [111]	Cross-sectional	155 women with SLE or rheumatoid arthritis	HCQ was associated with lower cholesterol (*P* < 0.001), triglycerides (*P* < 0.001) and LDL (*P* < 0.001), irrespective of concomitant steroids
2.	Kavanaugh et al. [112]	Pilot RCT	17 SLE patients	HCQ was associated with a significant reduction in total cholesterol in patients with SLE
3.	Pons-Estel et al. [38]	Prospective cohort	256 patients with LN	Lower LDL-cholesterol was seen in patients receiving antimalarials (*P* = 0.016)
4.	Chong et al. [55]	Cross-sectional study	100 patients with LN	HCQ was associated with lower levels of total cholesterol (*P* = 0.025) and LDL (*P* = 0.045)
5.	Meng et al. [113]	RCT	72 SLE patients	Total cholesterol, triglycerides, low-density lipoprotein, and high-density lipoprotein were statistically different (*P* < 0.05) between the two groups
E. Pregnancy				
1.	Izmirly et al. [114]	Retrospective cohort	257 pregnancies of anti-SSA/Ro-positive mothers with SLE	HCQ was significantly associated with a decreased risk of cardiac manifestations of neonatal lupus (OR, 0.23; 95% CI 0.06–0.92; *P* = 0.037)
2.	Moroni et al. [56]	Prospective study	71 pregnancies in 61 women with LN	Among pregnant women with lupus nephritis, HCQ users had an 85% reduction in the odds of having a small-for-gestational-age baby (OR 0.15, 95% CI 0.03–0.77; *P* = 0.0023) [56]
3.	Seo et al. [115]	Retrospective cohort	151 pregnancies in 122 SLE patients	HCQ was associated with lower risk of preeclampsia (OR 0.11, 95% CI 0.02–0.67)
F. Disease Flares and steroid minimization/withdrawal				
1.	Rothfield et. [116]	Retrospective study	43 SLE patients	HCQ reduced the required dose of steroids (*P* < 0.05) and risk of flares (*P* < 0.05)
2.	Pons-Estel et al. [38]	Prospective cohort (LUMINA)	256 patients with LN	Hydroxychloroquine-recipients received lower mean glucocorticoid doses than non-recipients (*P* = 0.025)
3.	Jorge et al. [95]	Retrospective cohort	342 patients with SLE	HCQ dose of ≤ 5 mg/kg/day was associated with an increased risk of lupus flares (adjusted OR 1.98, 95% CI 1.03–3.79)
4.	Fasano et al. [117]	Prospective cohort	154 SLE patients	Longer duration of HCQ therapy was associated with a reduced risk of disease flare (HR 0.84, 95% CI 0.72–0.98; *P* = 0.03) after steroid withdrawal
5.	Almeida-Brasil et al. [118]	Prospective cohort	1460 patients with SLE	Higher risk of SLE flare after HCQ reduction (HR 1.20, 95% CI 1.04–1.38) or HCQ discontinuation (HR 1.56, 95% CI 1.31–1.86), compared to HCQ maintenance
6.	Zen et al. [119]	Prospective cohort	513 SLE patients (270 with LN)	Patients treated with HCQ had a reduced risk of flares after withdrawal of immunosuppression (OR 0.194, 95% CI 0.038–0.978; *P* = 0.047)

*CI* confidence interval, *GC* glucocorticoids, *HCQ* hydroxychloroquine, *HR* hazard ratio, *IQR* Interquartile range, *LN* lupus nephritis, *MLN* Membranous Lupus Nephritis, *OR* odds ratio, *PCP* Pneumocystis pneumonia, *RCT* randomized controlled trial, *RR* relative risk, *SLICC* Systemic Lupus International Collaborating Clinics, *SLE* systemic lupus erythematosus

A recent systematic review concluded that HCQ was a safe and effective drug for proteinuria reduction, in addition to existing supportive treatment for IgA and could be an alternative, or an add-on treatment for those with inadequate response to immunosuppression [101]. Nevertheless, it would be premature to recommend routine use of HCQ in IgA nephropathy, until additional data from RCTs, or from large observational studies with long-term follow-up are available. Moreover, studies so far have mainly been conducted in the Chinese population, and therefore, lack of generalizability may be a problem. It is also unclear if the use of HCQ can delay progression to ESKD. Research on the use of HCQ for membranous nephropathy, Alport’s disease and other kidney diseases is still in the preliminary stages, and further studies are required to better understand its role in these conditions.

In conclusion, HCQ may have applications in nephrology practice beyond lupus nephritis; however, given the limitations of the evidence included in this narrative review, a broader routine use of HCQ cannot be recommended at present. There is an urgent need for well-designed studies to evaluate its role in other kidney diseases. A more comprehensive understanding of its mechanism of action is also needed. With the emerging role of blood level monitoring, there is reason to hope that the long-term safety of HCQ use may improve. This will not only enable individualized drug dosing but will also provide a tool to ensure drug compliance. However, additional data are needed to elucidate clinically relevant thresholds and frequency of monitoring.
